# Septic pulmonary embolism with spontaneous bilateral pneumothorax complicating facial cellulitis in an immunocompetent patient: a rare case report

**DOI:** 10.1093/omcr/omag101

**Published:** 2026-06-15

**Authors:** Mahad Sadik Mukhtar, Abdisalam Ismail Hassan, Amal Nor Ali, Jama Farah Abdillahi, Naima Awil Dahir, Ishak Ahmed Abdi, Abdirahim Ali Nor Adam, Said Abdirahman Ahmed, Abdijalil Abdullahi Ali, Ismail Gedi Ibrahim

**Affiliations:** Pulmonology Department, Mogadishu Somali-Türkiye Recep Tayyip Erdoğan Training and Research Hospital, Mogadishu, Somalia; Faculty of Medicine and Health Sciences, Jamhuriya University of Science and Technology, Mogadishu, Somalia; Pulmonology Department, Mogadishu Somali-Türkiye Recep Tayyip Erdoğan Training and Research Hospital, Mogadishu, Somalia; Pulmonology Department, Mogadishu Somali-Türkiye Recep Tayyip Erdoğan Training and Research Hospital, Mogadishu, Somalia; Pulmonology Department, Mogadishu Somali-Türkiye Recep Tayyip Erdoğan Training and Research Hospital, Mogadishu, Somalia; Cardiology Department, Mogadishu Somali-Türkiye Recep Tayyip Erdoğan Training and Research Hospital, Mogadishu, Somalia; Infectious Department, Mogadishu Somali-Türkiye Recep Tayyip Erdoğan Training and Research Hospital, Mogadishu, Somalia; Cardiology Department, Mogadishu Somali-Türkiye Recep Tayyip Erdoğan Training and Research Hospital, Mogadishu, Somalia; Cardiovascular Surgery Department, Mogadishu Somali-Türkiye Recep Tayyip Erdoğan Training and Research Hospital, Mogadishu, Somalia; Radiology Department, Mogadishu Somali-Türkiye Recep Tayyip Erdoğan Training and Research Hospital, Mogadishu, Somalia

**Keywords:** facial cellulitis, septic pulmonary embolism, pneumothorax, *Staphylococcus aureus*, Hydropneumothorax, low-resource settings, chest tube drainage

## Abstract

Background: A 20-year-old previously healthy male presented with progressive left-sided facial cellulitis, followed by respiratory deterioration. Imaging revealed SPE with bilateral pneumothoraces, more pronounced on the left side, requiring bilateral chest tube insertion. Blood cultures confirmed *Staphylococcus aureus*, and targeted antibiotic therapy led to clinical improvement. The patient recovered fully following multidisciplinary management. Discussion: This case demonstrates a rare progression of facial cellulitis to SPE and bilateral pneumothorax in an immunocompetent adult without traditional risk factors. Conclusion: Clinicians should consider thoracic complications in cellulitis patients with respiratory symptoms. Early imaging, microbiological evaluation, and prompt multidisciplinary management are critical, especially in low-resource settings.

## Introduction

Cellulitis is an acute bacterial infection of the skin and subcutaneous tissues, characterized by the absence of abscess or purulent discharge. It typically presents as a poorly defined, warm, erythematous area associated with edema and tenderness on palpation [1]. The most common causative organisms are group A *Streptococcus* (*Streptococcus pyogenes*) and *S. aureus*, including both methicillin-sensitive (MSSA) and methicillin-resistant (MRSA) strains [2]. These pathogens usually cause a localized, self-limiting infection that responds well to systemic antibiotic therapy [3].

Septic pulmonary embolism (SPE) is a serious infectious condition resulting from the hematogenous spread of infected emboli to the pulmonary vasculature. It may lead to peripheral pulmonary nodules, lung abscesses, and, in rare cases, pneumothorax (PTX) [4]. SPE is commonly associated with right-sided infective endocarditis, infected indwelling catheters, liver abscesses, skin and soft tissue infections, septic thrombophlebitis, Lemierre’s syndrome, dental infections, cardiac devices, and deep vein thrombosis [4, 5].

Although SPE is frequently linked to conditions such as right-sided endocarditis and intravenous access-related infections, its occurrence secondary to facial cellulitis in otherwise healthy individuals is extremely rare.

We present a rare case of left-sided facial cellulitis in a healthy young adult, complicated by *S. aureus* septic pulmonary embolism and bilateral large-volume pneumothoraces, more pronounced on the ipsilateral (left) side. This case highlights an unusual anatomical and pathophysiological progression of a common infection into a life-threatening systemic condition, emphasizing the importance of early recognition, multidisciplinary management, and awareness of such complications, particularly in low-resource settings.

## Case presentation

A 20-year-old previously healthy Somali male presented to the emergency department with a five-day history of progressive left-sided facial swelling and high-grade fever, followed by three days of shortness of breath and tachycardia.

On examination, his vital signs were blood pressure 130/85 mmHg, heart rate 118 beats per minute, respiratory rate 18 breaths per minute, and oxygen saturation 94% on room air. Physical examination revealed marked edema of the left face, particularly involving the lips and submandibular region, with associated erythema and tenderness. He had no known past medical history.

A superficial ultrasound of the face demonstrated diffuse soft tissue edema and inflammatory changes, along with multiple microabscesses, predominantly involving the left facial region, including the lips and submandibular area.

Laboratory investigations on admission revealed a markedly elevated white blood cell count of 20.56 × 10^9^/l and a C-reactive protein (CRP) level of 316.9 mg/l, indicating a severe systemic inflammatory response. Liver enzymes were mildly elevated with an AST of 125 U/l and ALT of 51 U/l. Serum albumin was 32 g/l. Electrolyte analysis showed hyponatremia (131 mmol/l) and hyperkalemia (6.31 mmol/l). Corrected calcium was 1.98 mmol/l, and uric acid was 95 μmol/l. Hemoglobin was 14.6 g/dl, and the platelet count was 237 × 10^9^/l.

Blood cultures were obtained at admission, and empiric broad-spectrum antibiotics were initiated. On Day 4, the patient developed worsening dyspnea and hypoxia. A chest CT scan revealed multiple peripherally distributed pulmonary nodules, some with a feeding vessel sign and cavitation, along with wedge-shaped subpleural lesions, consistent with septic pulmonary embolism (SPE) (Fig. 1). Transthoracic echocardiography was performed and showed no evidence of valvular vegetations or features suggestive of infective endocarditis.

**Figure 1:**
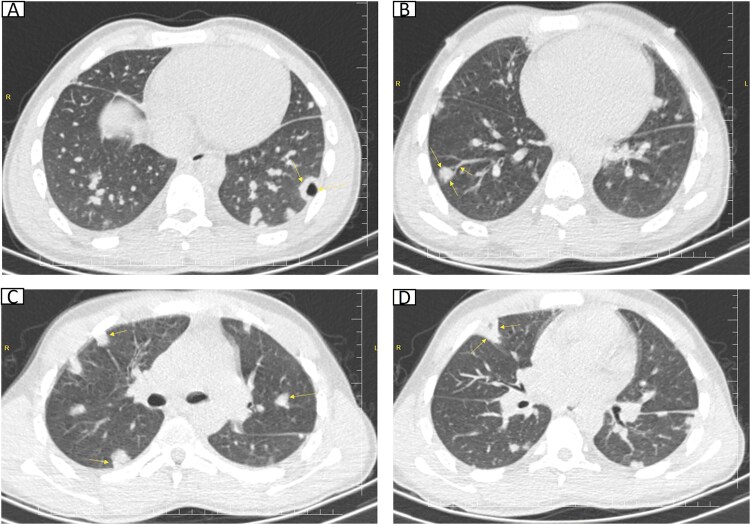
Axial chest CT images demonstrate characteristic features of septic pulmonary embolism. (A) Shows a subpleural cavitary lesion (arrow), representing necrosis of a peripheral embolic focus. (B) Demonstrates a pulmonary nodule with a feeding vessel sign (arrow), indicating a direct vascular source of embolization. (C) Reveals multiple peripherally distributed nodules (arrows), consistent with hematogenous spread. (D) Shows a wedge-shaped subpleural lesion (arrow), suggestive of pulmonary infarction secondary to embolic occlusion.

The blood culture subsequently yielded *S. aureus*, sensitive to vancomycin, clindamycin, and meropenem. Targeted antimicrobial therapy was initiated on Day 8 following availability of culture and sensitivity results.

The interval reflects the time required for organism identification and antimicrobial susceptibility testing.

Despite treatment, the patient developed bilateral large-volume pneumothoraces, more severe on the left side, resulting in near-complete collapse of the left lung. A mild pleural effusion was also noted. The thoracic surgery team was consulted, and bilateral chest tubes were inserted.

Follow-up chest radiographs showed a pneumohydrothorax and collapsed lung, with bilateral chest tubes in situ. Subsequent imaging demonstrated resolution of the pneumothoraces and pleural effusions, along with regression of the pulmonary nodules (Fig. 2). The chest tubes were removed on Day 20, and the patient was discharged in stable condition. At follow-up on Day 31, he remained well with no residual symptoms.

**Figure 2:**
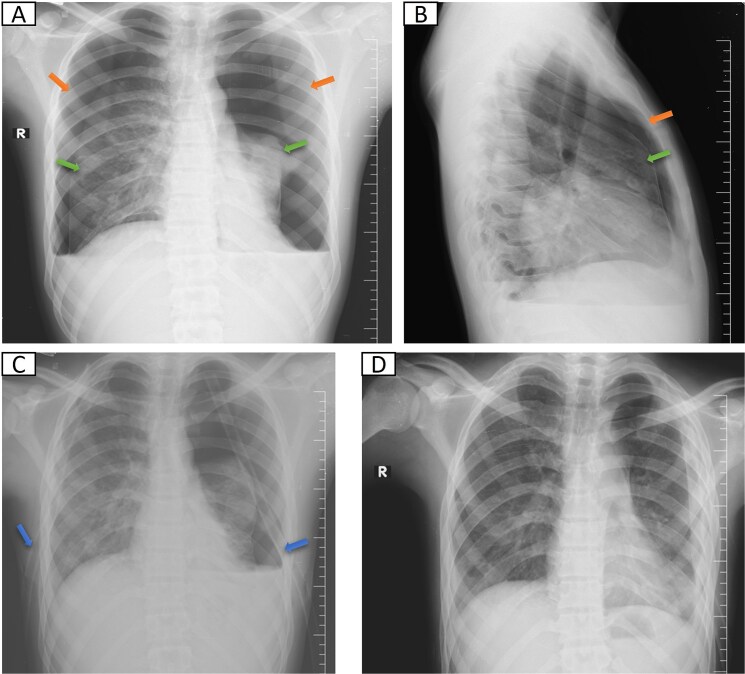
Chest radiographs illustrate the progression and resolution of thoracic complications. Frontal and lateral views (A and B) demonstrate a pneumohydrothorax (orange arrow) and associated lung collapse (green arrow). Image (C) shows bilateral chest tubes in situ (blue arrows) following intervention. Follow-up radiograph (D) demonstrates complete resolution of the pneumothorax with re-expansion of both lungs and improvement of pleural pathology.

## Discussion

This case highlights a rare but clinically important progression of left-sided facial cellulitis to septic pulmonary embolism (SPE), followed by bilateral pneumothorax in a previously healthy adult. While facial cellulitis is normally a limited soft tissue infection with a good prognosis, this case demonstrates how, under certain conditions, hematogenous spread of bacteria such as *S. aureus* can lead to life-threatening systemic and thoracic complications.

Facial cellulitis is a relatively uncommon presentation of soft tissue infection, most commonly caused by *S. pyogenes* and *S. aureus*, both of which are part of normal flora but can act as opportunistic pathogens [6]. Methicillin-resistant strains of *S. aureus* are associated with increased virulence, antibiotic resistance, and a higher risk of complications [7]. Although cellulitis typically involves the lower extremities, it may also affect the face, abdominal wall, and upper extremities [7]. In contrast to orbital or preseptal cellulitis, which are more commonly associated with streptococcal species, our case involved the cheek and submandibular region, representing an atypical anatomical distribution.

Septic pulmonary embolism (SPE) is an uncommon but serious condition caused by infected thrombi entering the pulmonary circulation, leading to peripheral pulmonary nodules, infarction, cavitation, and occasionally pleural complications such as pneumothorax [4]. Early administration of empiric antibiotic therapy targeting common pathogens such as *S. aureus* and *Streptococcus* species is essential for optimal outcomes [8].

The development of pneumothorax in SPE is primarily related to peripheral, subpleural septic embolic lesions. These emboli can undergo necrosis and cavitation, forming thin-walled cavities adjacent to the pleural surface. Subsequent rupture of these cavitary lesions into the pleural space may result in pneumothorax. In our case, chest CT demonstrated peripherally distributed nodules with cavitation and wedge-shaped subpleural lesions, which anatomically predispose to pleural rupture and explain the occurrence of bilateral pneumothoraces [9].

The lung parenchyma becomes structurally compromised due to these necrotizing and cavitating lesions, making it more susceptible to alveolopleural fistula formation and pneumothorax even at relatively low airway pressures. This vulnerability is particularly relevant in the presence of extensive peripheral disease, as seen in our patient. In mechanically ventilated patients, a lung-protective strategy with low tidal volumes and minimized airway pressures is recommended, along with prompt pleural drainage when pneumothorax occurs [9].

Pneumothorax is a rare but potentially catastrophic complication of SPE and may occur bilaterally. A bronchopleural or alveolopleural fistula may develop secondary to rupture of cavitary lesions caused by septic emboli, particularly those associated with *S. aureus* [4, 9].

Secondary pneumothorax occurs in a small proportion of SPE cases (approximately 3.4%) and is associated with a high in-hospital mortality rate [10]. The mechanisms include cavitation of peripheral nodules, aseptic necrosis from vascular occlusion, and secondary infection leading to destruction of lung parenchyma. Importantly, while right-sided infective endocarditis is a common source of SPE, it was excluded in our case by normal echocardiographic findings, further emphasizing an alternative hematogenous source.

Cellulitis risk factors include any disruption of the skin barrier, such as trauma, surgical wounds, intravenous punctures, fissures, insect or animal bites, and pre-existing skin infections. Patients with comorbidities such as diabetes mellitus, venous insufficiency, peripheral arterial disease, and lymphedema are at increased risk [2]. Imaging plays a crucial role in diagnosis; MRI is superior for soft tissue evaluation, whereas CT is particularly useful for detecting deep infection and associated complications [11].

A comparable case of bilateral orbital cellulitis complicated by SPE and subsequent bilateral pneumothorax has been reported in the pediatric population, although it remains exceedingly rare [12]. However, that case differs significantly from ours in terms of patient age, anatomical site of infection, and clinical evolution. To our knowledge, no previous report has described an adult with left-sided facial cellulitis progressing to SPE and bilateral pneumothorax, making this case unique in both anatomical and demographic context.

Furthermore, most reported cases of SPE-associated pneumothorax are linked to intravenous drug use, right-sided infective endocarditis, or indwelling catheter-related infections, none of which were present in our patient [2, 4, 5]. The absence of these traditional risk factors highlights the unpredictable and potentially severe course of even localized soft tissue infections, particularly those caused by virulent organisms such as *S. aureus*. This underscores the importance of maintaining a high index of suspicion, especially in low-resource settings where delayed diagnosis may worsen outcomes.

Our patient, who was previously healthy and immunocompetent, illustrates that facial cellulitis can, in rare cases, progress to a life-threatening thoracic complication. This emphasizes the importance of close monitoring, early imaging, and multidisciplinary management in preventing and addressing severe complications.

## Conclusion

This case illustrates a rare but life-threatening progression of facial cellulitis to septic pulmonary embolism and bilateral pneumothorax in an immunocompetent adult. Although typically localized, facial cellulitis may rarely lead to severe systemic complications. The development of respiratory symptoms should prompt early chest imaging and comprehensive evaluation. Timely microbiological diagnosis and multidisciplinary management are essential to improve outcomes, particularly in low-resource settings where delays in diagnosis and treatment may adversely affect prognosis.

## Data Availability

The data supporting the findings of this study are available from both corresponding authors upon reasonable request.
